# Whole exome sequencing identifies a novel mutation in *Annexin A4* that is associated with recurrent spontaneous abortion

**DOI:** 10.3389/fmed.2024.1462649

**Published:** 2024-09-27

**Authors:** Qian Ye, Fa-Ying Liu, Xiao-Jian Xia, Xiao-Yong Chen, Li Zou, Hui-Min Wu, Dan-Dan Li, Chen-Nian Xia, Ting Huang, Ying Cui, Yang Zou

**Affiliations:** ^1^Department of Traditional Chinese Medicine, Jiangxi Maternal and Child Health Hospital, Nanchang, China; ^2^Key Laboratory of Women's Reproductive Health of Jiangxi Province, Jiangxi Maternal and Child Health Hospital, Nanchang, China; ^3^Key Research Unit of Female Reproduction with Integrated Chinese and Western Medicine of Jiangxi Province, Jiangxi Maternal and Child Health Hospital, Nanchang, China; ^4^Central Laboratory, Jiangxi Maternal and Child Health Hospital, Nanchang, China; ^5^Quality Control Office, Ganzhou People's Hospital, Ganzhou, China; ^6^Graduate School of Clinical Medicine, Jiangxi University of Traditional Chinese Medicine, Nanchang, China

**Keywords:** *ANXA4* mutation, recurrent spontaneous abortion, cell invasion, migration, adhesion

## Abstract

**Background:**

Recurrent spontaneous abortion (RSA) is a multifactorial disease, the exact causes of which are still unknown. Environmental, maternal, and genetic factors have been shown to contribute to this condition. The aim of this study was to investigate the presence of mutations in the *ANXA4* gene in patients with RSA.

**Methods:**

Genomic DNA was extracted from 325 patients with RSA and 941 control women with a normal reproductive history for whole-exome sequencing (WES). The detected variants were annotated and filtered, and the pathogenicity of the variants was predicted through the SIFT online tool, functional enrichment analyses, Sanger sequencing validation, prediction of changes in protein structure, and evolutionary conservation analysis. Furthermore, plasmid construction, Western blotting, RT–qPCR, and cell migration, invasion and adhesion assays were used to detect the effects of *ANXA4* mutations on protein function.

**Results:**

An *ANXA4* mutation (p.G8D) in 1 of the 325 samples from patients with RSA (RSA-219) was identified through WES. This mutation was not detected in 941 controls or included in public databases. Evolutionary conservation analysis revealed that the amino acid residue affected by the mutation (p.G8D) was highly conserved among 13 vertebrate species, and the SIFT program and structural modeling analysis predicted that this mutation was harmful. Furthermore, functional assays revealed that this mutation could inhibit cell migration, invasion and adhesion.

**Conclusion:**

Our study suggests that an unreported novel *ANXA4* mutation (p.G8D) plays an important role in the pathogenesis of RSA and may contribute to the genetic diagnosis of RSA.

## 1 Introduction

Recurrent spontaneous abortion (RSA) is a common complication in women of childbearing age during pregnancy, with an incidence rate ranging from 1 to 5% (1).

The number of miscarriages required for a diagnosis of RSA is controversial because the risk of another miscarriage after two consecutive miscarriages is similar to that after three consecutive miscarriages, and recent studies have suggested that predicting pregnancy loss after two consecutive miscarriages is more reasonable (2, 3). The American Reproductive Association defines recurrent spontaneous abortion as two or more consecutive miscarriages (4). Obviously, the pathogenesis of RSA is complex. To date, the potential risk factors for RSA reported include genetic, immunologic, anatomic, and infectious anomalies (5–10). However, approximately 50% of RSAs are unexplained or poorly understood (11).

For almost all human diseases, individual susceptibility is influenced by genetic variations to some degree (12). In recent years, whole-genome sequencing (WGS) and whole-exome sequencing (WES) have proven to be powerful new approaches for identifying disease-associated variants across the full minor allele frequency (MAF) spectrum in animals and humans (13–15). Genome-wide screening has shown that siblings of patients with RSA have a greater risk of miscarriage (16), Variants associated with RSA have been detected in genes involved in the immune response (*IFNG, IL10, KIR2DS2, KIR2DS3, KIR2DS4, MBL,* and *TNF*), coagulation (*F2, F5, PAI-1,* and *PROZ*), metabolism (*GSTT1* and *MTHFR*), hormonal regulation (*ESR1* and *ADRB2*), angiogenesis (*NOS3* and *VEGFA*), and endometrial and placental function (*ENOS* and *ACE*) (17–19). Moreover, several studies have shown that rare variants play crucial roles in multiple human diseases, including RSA (20–22). However, few studies have confirmed that rare variants may play certain roles in the pathogenesis of RSA at the whole-exome/genome scale.

Annexin A4 (*ANXA4*) is a cytosolic calcium-binding protein with four repeat domains, each containing one calcium-binding site (CBS), and belongs to a ubiquitous family of Ca^2+^-dependent membrane-binding proteins thought to be involved in membrane trafficking and membrane organization within cells (23). Previous studies have shown that *ANXA4* modulates membrane permeability and membrane trafficking, participates in cellular growth and apoptosis, and enhances tumor invasion (24). *ANXA4* has been shown to be upregulated in various clinical epithelial tumors, including colorectal, pancreatic, ovarian, breast, and prostate cancers (24). Furthermore, a previous study revealed that the *ANXA4* protein is localized to the glandular and luminal epithelium of the human endometrium and is present at high levels throughout the menstrual cycle, except during the early secretory (ES) phase (25).

In this study, we used WES to explore the potential involvement of rare variants in RSA risk in 325 patients with RSA. Among these rare variants, a novel missense mutation in the *ANXA4* gene (c.23G > A, p.G8D) was detected in 1 patient with RSA (RSA-219). This variant was not found in either 941 controls or public databases. Functional assays revealed that this novel mutation could influence cell migration, invasion and adhesion, indicating that the *ANXA4* mutation p.G8D contributes to the pathogenesis of RSA in women of Chinese ethnicity.

## 2 Materials and methods

### 2.1 Clinical samples

A total of 325 Han Chinese RSA patients and 941 control individuals at the Maternal and Child Health Hospital of Jiangxi Province between August 2018 and October 2019 were enrolled in this study. The RSA patients had experienced more than 2 unexplained and consecutive spontaneous abortions at less than 10 weeks of gestation. Patients who had genital abnormalities; chronic hypertension; diabetes; liver, kidney, cardiovascular, and thyroid diseases; autoimmune diseases; or infectious diseases were excluded. The control group was composed of women who underwent induced abortions of a normal pregnancy at a gestational age matched to that of the RSA group. The individuals in the control group were not treated with any drugs and had no pregnancy risk. The basic information of our study population is summarized in Table 1.

**Table 1:** The basic clinical features of RSA patients and controls.

Characteristic		RSA patients	Controls
No. of samples		325	941
Age at diagnosis (years)		30.4 ± 5.27	29.4 ± 5.07
Gravidity		4.3 ± 1.38	1.9 ± 1.72
	<2	0	347
	2–4	232	556
	5–7	86	38
	>7	7	0
Parity		0.5 ± 0.64	NA
	0	189	
	1	102	
	≥2	34	
Number of spontaneous abortions		2.4 ± 0.74	NA
	2	237	
	3	57	
	4	20	
	≥5	11	

NA, not available; RSA, recurrent spontaneous abortion. All data are presented as mean ± SEM.

All patients signed an informed consent form before being enrolled in the study, and this study was approved by the Ethics Committee of Jiangxi Provincial Maternal and Child Health Hospital (EC-KY-202149).

### 2.2 Exome sequencing and data analysis

Genomic DNA was extracted from the peripheral blood of each sample with the AxyPrep Blood Genomic DNA Miniprep Kit (Axygen Scientific, Inc., 33,210 Central Avenue, Union City, CA 94587, United States) according to the instructions provided by the manufacturer. DNA quality and concentration were determined with a NanoDrop-1000 spectrophotometer (Thermo Fisher, United States) and gel electrophoresis, respectively. Exome capture was performed with a BGI Exome V4 (59 Mb) Kit (BGI, China) according to the manufacturer’s protocols. DNA sequencing was performed on BGISEQ-500 sequencers (BGI, China) in high-output mode with 100 bp paired-end reads. Exome data were mapped to the human reference genome GRCh37 using the Burrows–Wheeler Aligner (BWA-MEM, version 0.6.2) (26). The credibility and quality of single-nucleotide polymorphisms (SNPs) and small insertions/deletions (InDels) were detected with the Genome Analysis Tool Kit (GATK, version 3.7) (27). ANNOVAR was used for the annotation and classification of SNPs and InDels, respectively (28). Variants with a read depth > 20 were extracted for further analysis. Finally, the variants identified through the above pipeline were filtered to eliminate benign variants with minor allele frequency (MAF) ≥1% in the dbSNP database,^1^ 1,000 Genomes database,^2^ Exome Aggregation Consortium (ExAC, http://exac.broadinstitute.org/) and BGI’s in-house databases containing exome data from >100,000 subjects (29, 30). We ranked the genes and their potential for damage using the online prediction program SIFT to analyze whether these variants were potentially pathogenic. Variants that received lower SIFT scores (typically ≤0.05), indicating a greater likelihood of affecting protein function, were predicted to be damaging.

### 2.3 Validation by Sanger sequencing

Sanger sequencing was used to verify the mutation in the gene of interest in RSA patients. The polymerase chain reaction (PCR) primer was designed according to the mutation site in the *ANXA4* gene with Primer 3 (F: 5’-GGCCTCGAAGAACTTCTGCT-3′, R: 5’-TGGGCATCTTCCATGGCATT-3′). PCR amplification was performed using the rTaq enzyme (Takara Biotechnology, Dalian, China) with 50 ng of genomic DNA as a template. PCR was performed with an ABI 2720 thermocycler (Applied Biosystems, Waltham, MA), and the reaction conditions were as follows: the first step was 94°C for 5 min, followed by 30 cycles of denaturation at 94°C for 30 s, annealing at 52°C for 30 s, and extension at 72°C for 30 s and a final extension at 72°C for 10 min. The amplified PCR products were subjected to 1% agarose gel electrophoresis and then purified from the gel with a DNA purification kit (Tiangen, Beijing, China). The purified PCR products were sequenced with an ABI 3730 Automatic Capillary DNA Sequencer (Applied Biosystems, Waltham, MA). The obtained DNA sequences were analyzed using DNASTAR Lasergene version 7.2 software (DNASTAR, Inc., Madison, WI, United States) with the reference sequence from the NCBI database.

### 2.4 Evolutionary conservation analysis of the rare *ANXA4* variant (p.G8D)

To predict the potential pathogenicity of the mutation, evolutionary conservation analysis was performed by aligning the amino acid sequences of *ANXA4* proteins from 13 species obtained from the GenBank database,^3^ including *Human* (NP_001144.1), *Chimpanzee* (XP_016804171.1), *Pongo abelii* (XP_002811982.2), *Green monkey* (XP_007968539.1), *Nomascus leucogenys* (XP_030683909.1), *Mouse* (NP_001318049.1), *Rat* (NP_077069.3), *Cattle* (NP_001001440.2), *Pig* (NP_001161111.1), *Dog* (NP_001003039.2), *Horse* (XP_023474726.1), *Rabbit* (XP_017196184.1) and *Goat* (XP_017910949.1). Evolutionary conservation analysis was performed using MEGA4 software.

### 2.5 Protein structural modeling

The modeling of the protein template between the reference and the modified (p.G8D) mutation of the *ANXA4* gene was conducted using the SWISS-MODEL repository database.^4^ Then, we employed the Chimera 1.14rc package to concurrently compare the protein models.

### 2.6 Plasmid construction

Wild-type and mutant *ANXA4* (NM_001153.5) were obtained from GeneCreate Biological Engineering Co., Ltd. (Wuhan, China). Human *ANXA4* cDNA was inserted into the pcDNA3.1 vector to generate the wild-type plasmid. The mutant plasmid was generated with the wild-type plasmid as the template using a KOD-Plus-Mutagenesis Kit (Toyobo, Osaka, Japan). All the plasmids were confirmed by Sanger sequencing.

### 2.7 Cell culture and transfection

The THESCs cell line (human endometrial stromal cell line) was obtained from ATCC (Manassas, VA, http://www.lgcstandards-atcc.org). The cells were cultured in DMEM/F12 medium (HyClone, GE, United States) supplemented with 10% fetal bovine serum (FBS) (SA211.02, Minhai Bioengineering Co., Ltd., Lanzhou, China) and 1% penicillin–streptomycin (PS) (P1400, Beijing Solarbio Science & Technology Co., Ltd., Beijing, China) in an incubator at 37°C with a humidified atmosphere of 5% CO2.

The wild-type or mutant *ANXA4* plasmids were transfected into THESCs with FuGENE HD transfection reagent (Promega, E2311) according to the manufacturer’s instructions. Briefly, 1 × 10^5^ THESCs were seeded in 6-well plates and cultured for 24 h. The medium in the 6-well plate was subsequently discarded, and 800 μL of DMEM (without FBS or antibiotics) was added to each well. A total of 100 μL of DMEM (without FBS or antibiotics), 2 μg of plasmid and 5 μL of FuGENE HD transfection reagent were mixed together, and the mixture was incubated for 10 min at room temperature. Finally, the transfection mixture was added to the cells gently and incubated in an incubator with 5% CO_2_ at 37°C for 2 h. Then, 1 mL of DMEM with 10% FBS was added to each well, and the cells were cultured in an incubator with 5% CO_2_ at 37°C.

### 2.8 Western blotting

Protein expression was detected by Western blotting analysis. Forty-eight hours after transfection, the cells were treated with cell lysis buffer (Applygen Technologies Co., Ltd., Beijing). The protein concentration was determined by using a bicinchoninic acid (BCA) protein analysis kit (Thermo Fisher Biochemical, Beijing). The protein samples (20–30 μg) were separated by 10% sodium dodecyl sulfate polyacrylamide gel electrophoresis (SDS–PAGE), transferred to polyvinylidene difluoride (PVDF) membranes (Merck KGaA, United States), and blocked in 5% skim milk for 1 h at room temperature. The membranes were subsequently incubated with the following primary antibodies at 4°C overnight: anti-*ANXA4* (1:1000, TA803051; Origene, China) and anti-*β*-actin (1,2000, sc-47778; Santa Cruz). After being washed with 1× TBST three times, the membranes were incubated with the corresponding secondary antibodies for 2 h at room temperature. Protein bands were visualized using luminol reagent (sc-2048; Santa Cruz Biotechnology Company, United States) or enhanced chemiluminescence reagent (Santa Cruz Biotechnology Company, United States) with a ChemiDoc XRS instrument (Bio-Rad Laboratories, CA, United States). Relative protein expression levels were measured using ImageJ software (NIH).

### 2.9 RNA isolation and real-time quantitative PCR

RT–qPCR was used to assess the mRNA expression of *ANXA4*. The quality and concentration of total RNA were confirmed with a Nanodrop 2000 instrument (Thermo Scientific, United States), followed by extraction from treated cells using TRIzol reagent (Invitrogen, United States). Five hundred nanograms of RNA was reverse transcribed to synthesize cDNA (20 μL) using a PrimeScript 1st Strand cDNA Synthesis Kit (TaKaRa, Japan) according to the manufacturer’s protocol. Then, qPCR was performed with SYBR Green PCR Master Mix (TaKaRa, Japan) on an ABI 7500 thermocycler (Applied Biosystems, Waltham, MA). *ANXA4* was used as the target gene, and *GAPDH* was used as an internal reference. The primer sequences for both genes were designed and validated for specificity. The primer sequences used for RT–qPCR were as follows: *ANXA4*-F (5’-TGCCCTGCTGAGCTGGACTT-3′) and *ANXA4*-R (5’-AAAGCTGCTCAGGACCATGT-3′); *GAPDH*-F (5’-GGAGCGAGATCCCTCCAAAAT-3′) and *GAPDH*-R (5′- GGCTGTTGTCATACTTCTCATGG-3′). The following qPCR conditions were used: initial denaturation at 95°C for 2 min, followed by 30 cycles of denaturation at 95°C for 15 s, annealing at 60°C for 10 s, and extension at 72°C for 15 s and a final extension at 72°C for 10 min. The 2^−ΔΔCt^ method was used to calculate and normalize the relative expression of *ANXA4* to that of *GAPDH*.

### 2.10 Cell migration and invasion assays

The migration and invasion capabilities of THESCs transfected with wild-type or mutant *ANXA4* plasmids were assessed with Transwell assays (Corning, Toledo, OH, United States).

For the migration analysis, after 48 h of transfection, the cells (3 × 10^5^/ml) were collected, resuspended in DMEM/F12 without FBS, and then inoculated into the upper Transwell chamber, while the lower chamber was filled with 500 μL of DMEM/F12 supplemented with 20% FBS. After incubation for 24 h, the upper chambers were washed, fixed with methanol for 20 min, stained with 1% crystal violet dye solution for 30 min at room temperature, and washed with phosphate-buffered saline (PBS) several times. The chamber membranes were then photographed via light microscopy (IX71, OLYMPUS, Japan), and the number of migratory cells on the lower surface was counted in five random fields to assess the migration capability.

For invasion analysis, a total of 4 × 10^5^ cells were seeded into the upper Transwell chamber, after which the upper chamber was precoated with Matrigel matrix (3.5 mg/mL) (BD Biosciences, United States). After 48 h, the same protocol as in the migration analysis was performed.

### 2.11 Cell adhesion assay

Briefly, after 48 h of transfection, a total of 4 × 10^3^ cells/well were seeded in a 96-well plate, which was pretreated with 50 μL/well of Matrigel matrix (BD Biosciences, United States) and incubated at 37°C for 30 min to allow polymerization. Then, the 96-well plates were incubated in an incubator with 5% CO_2_ at 37°C for 20 min, 40 min or 60 min. The plates were washed with PBS three times to remove nonadherent cells, and the adherent cells were fixed with methanol for 15 min and stained with 1% crystal violet dye solution for 30 min at room temperature. The numbers of adherent cells were counted using light microscopy (IX71, OLYMPUS, Japan).

### 2.12 Statistical analyses

All the statistical analyses were performed using the statistical analysis software SPSS 21.0. Data are expressed as the mean ± standard error of the mean (S.E.M.). An unpaired Student’s *t* test was used to evaluate the significant differences among the groups. Statistical significance was established at a *p* value of less than 0.05.

## 3 Results

### 3.1 Identification of one novel rare variant in *ANXA4* in one patient with RSA using WES

In total, 325 patients with RSA and 941 local women without RSA as controls underwent WES. WES performed on BGISEQ-500 sequencers achieved, on average, 21,266.42 Mb of raw bases. After removing low-quality reads, we obtained an average of 212,622,806 clean reads (21,255.71 Mb). The clean reads of each sample had high Q20 and Q30 values, indicating high sequencing quality. The average GC content was 52.14%. The chromosomal positions of the SNPs were based on the UCSC GRCh37/hg19 genome.

We obtained a total of 79,534 variants, including synonymous, missense, stop-loss, stop-gain, start-loss and splicing variants. The SnpEff tool^5^ was used to perform variant annotation and prediction. After excluding variants with MAFs greater than or equal to 1% in publicly available databases, e.g., dbSNP, 1,000 Genomes Project, ExAC and BGI’s in-house databases, 262 variants were retained. Using the *in silico* prediction algorithm SIFT, we selected candidate variants with potentially damaging impacts. The results showed that the *ANXA4* gene was prominent on the basis of its functional annotation related to cell migration and invasion and the pathogenicity predictions of its mutations.

**Figure 1:**
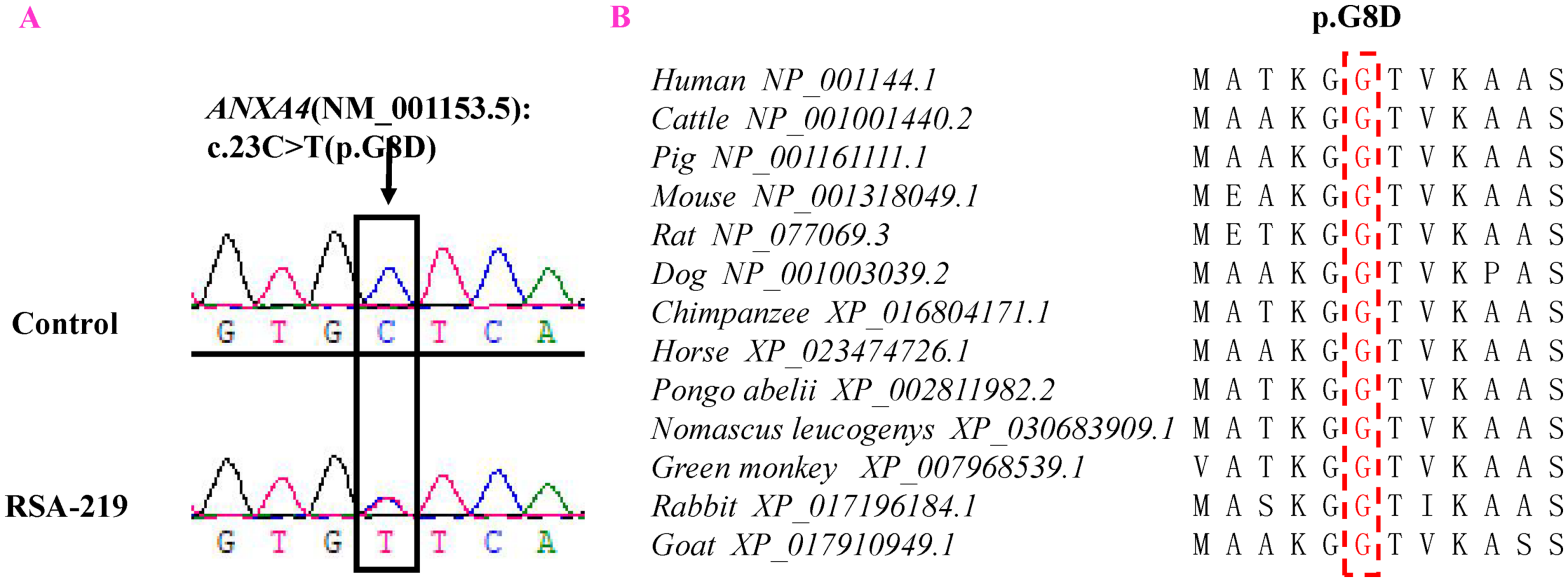
Representative sequencing electropherogram of the *ANXA4* variant (p.G8D). The arrow indicates the location of the mutation, and patient “RSA-219” harbored the *ANXA4* p.G8D variant **(A)**. Evolutionary conservation analysis of *ANXA4* p.G8D in vertebrate species **(B)**.

A novel mutation (c.23G > A, p.G8D) in the *ANXA4* gene (NM_001153.5) was detected in 1 of the 325 patients with RSA (RSA-219) but was not detected in either the 941 controls or public databases (dbSNP, 1,000 Genomes Project, ExAC and BGI in-house databases). Primers were designed for PCR to amplify the *ANXA4* fragment containing the rare variant, and Sanger sequencing was performed to validate this mutation (Figure 1A). The patient (RSA-219) was confirmed to harbor this mutation by Sanger sequencing. The *ANXA4* gene is located on chromosome 2p13.3 and consists of 22 exons. The mutation is positioned in the first exon.

### 3.2 Evolutionary conservation analysis and protein structural modeling

**Figure 2:**
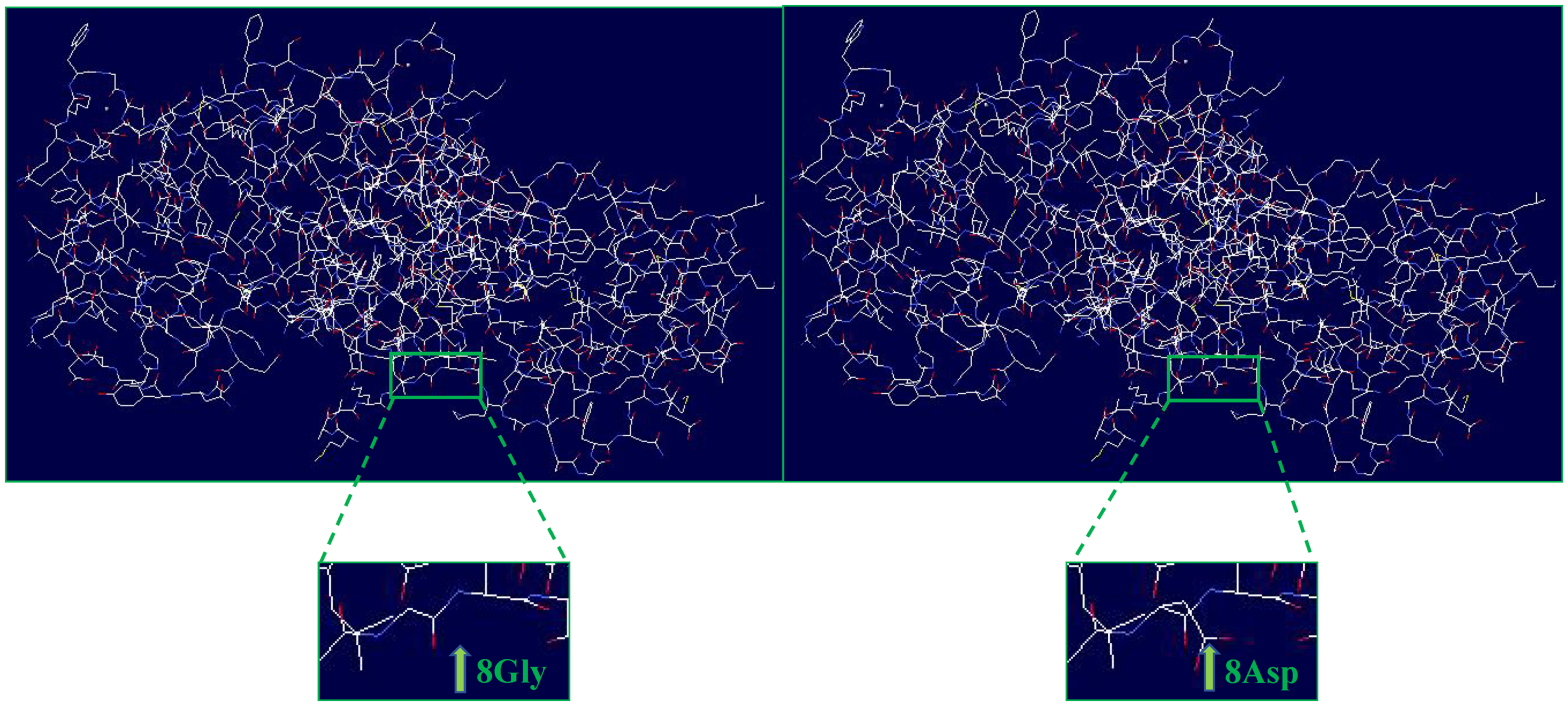
Structural differences between wild-type *ANXA4* (p.G8) and mutated *ANXA4* (p.D8) proteins. The protein structures of the wild-type and mutated *ANXA4* proteins were modeled based on the crystal model of the human *ANXA4* protein.

Evolutionary conservation analysis revealed that the amino acid residue affected by the p.G8D mutation was highly conserved among 13 vertebrate species (Figure 1B). Furthermore, we analyzed the changes in the 3D structure of *ANXA4* between the wild-type and mutant proteins using SWISS-MODEL. Protein structural modeling revealed that the p.G8D *ANXA4* mutation caused structural changes in the *ANXA4* protein (Figure 2).

### 3.3 A novel mutation in *ANXA4* (p.G8D) inhibits the migration, invasion and adhesion of THESCs

Annexins have been suggested to promote cellular migration, invasion and adhesion, and adhesion is an essential step required for blastocysts to adhere to the endometrium (31). Next, we examined whether the p.G8D mutation in *ANXA4* affects protein function.

**Figure 3:**
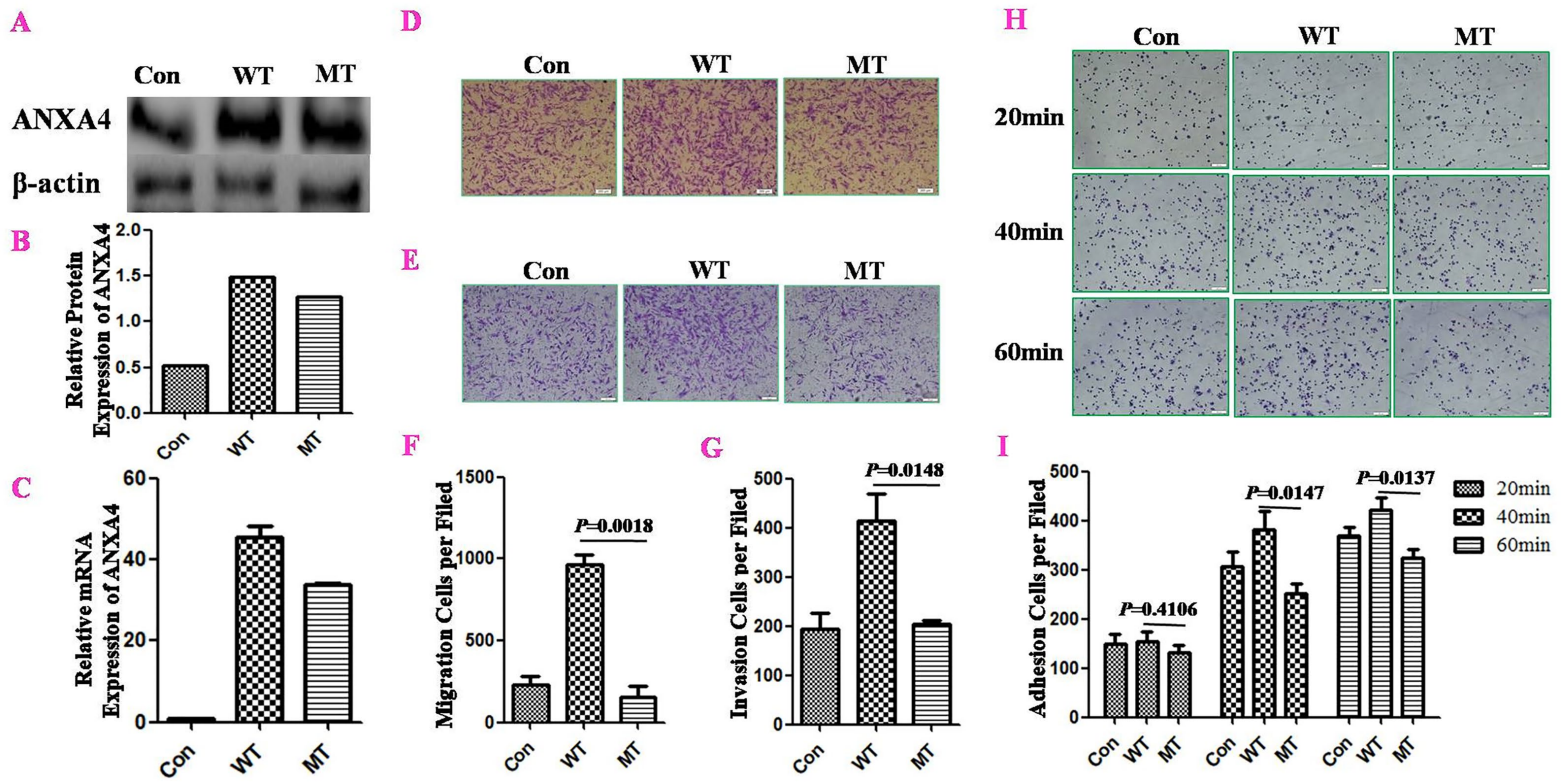
The *ANXA4* p.G8D mutation inhibited cell migration, invasion and adhesion in stably transfected THESCs. THESCs were cultured and transfected with *ANXA4* wild-type (WT) or mutant-type (MT) plasmids, while pcDNA3.1 was used as a control plasmid (Con). Twenty-four hours after transfection, G418 was used to screen stably transfected cells, and one individual clone was separated by dilution. *ANXA4* expression in stably transfected cells was confirmed by WB **(A,B)** and RT–qPCR **(C)**. The effects of *ANXA4* mutation on cell migration **(D,F)** and invasion **(E,G)** were determined by Transwell assays, and cell adhesion **(H,I)** was determined by Matrigel adhesion assays. These assays revealed that *ANXA4* p.G8D mutation inhibited cell migration, invasion and adhesion in stably transfected cells compared with *ANXA4* WT. Scale bars, 100 μm. The data are shown as the means ± SDs. *p* < 0.05 was considered to indicate a significant difference.

THESCs were cultured and transfected with *ANXA4* wild-type (WT) or mutant-type (MT) plasmids, and pcDNA3.1 was used as a control plasmid (Con). Twenty-four hours after transfection, G418 was used to screen stably transfected cells, and one individual clone was separated by dilution. The expression of *ANXA4* in stably transfected cells was confirmed by RT–qPCR and WB (Figures 3A–C).

Transwell assays were used to detect the effects of *ANXA4* WT/MT on migration and invasion. The results showed that MT *ANXA4* could inhibit both cell migration (*p* = 0.0018) (Figures 3D,F) and invasion (*p* = 0.0148) (Figures 3E,G) of stable transfected cells, compared to WT *ANXA4*.

We examined the effect of the *ANXA4* mutation on cell adhesion with a Matrigel adhesion assay. Compared with WT *ANXA4*, MT *ANXA4* inhibited cell adhesion, especially at 40 min (*p* = 0.0147) and 60 min (*p* = 0.0137) (Figures 3H,I).

These results indicated that the novel *ANXA4* mutation inhibited cell migration, invasion and adhesion.

## 4 Discussion

The main cause of abortion is implantation failure. Embryo implantation is a dynamic development process that requires a series of interactions between the blastocyst and the endometrium. The three prerequisites for successful implantation are an embryo with implantation ability, a receptive endometrium and simultaneous development of the embryo-endometrium. Many existing animal studies have confirmed that the uterus can keep the embryo in a viable but dormant state until the uterus is in an acceptable state for implantation, indicating that the proper regulation of the uterus by ovarian hormones mainly determines the success of implantation (32). Decidualization of the endometrium leads to corresponding vascular changes preparing for the invasion of human endometrial trophoblasts and the formation of a functional blood placenta; in other words, decidualization contributes to the functional transition of the endometrium from a nonreceptive state to a receptive state (33). An increasing amount of experimental and clinical data indicates that impaired or disrupted decidualization plays a role in the development of an unsuitable maternal–fetal interface. This situation has important clinical implications, including repeated implantation failures and recurrent pregnancy losses in the early stages of pregnancy, as well as several serious complications during late gestation (34). Studies have shown that in the presence of trophoblasts, the motility and invasive ability of decidualized endometrial stromal cells are enhanced (35), and trophoblast cell-derived CXCL12 has been shown to promote the expression and invasiveness of CXCR4 (the receptor of CXCL12) in decidual stromal cells isolated from early pregnancy (36). Many studies have shown that the coordinated migration and invasion of decidualized endometrial stromal cells in response to signals from embryo and trophoblast cells is the key to successful implantation. The ability of mature endometrial stromal cells to migrate and invade is increasingly recognized as the basis of intense tissue remodeling associated with endometrial regeneration, decidualization, embryo implantation, and trophoblast invasion (37, 38).

The annexin family is a class of proteins that are widely present in cell membranes and intracellular organs and are known mainly for their ability to bind phospholipids and their dependence on calcium ions. Given that the annexin family is highly expressed at the maternal–fetal interface and that both the endometrium and blastocyst undergo swift growth and differentiation throughout pregnancy, annexin is believed to perform various functions in both the placental tissues originating from the fetus and the decidual cells derived from the mother (39). Defective expression of endometrial ANXA2 may impair decidualization of endometrial stromal cells *in vitro* and *in vivo*, leading to the development of preeclampsia (40). Previous studies have demonstrated that the binding of antibodies and the apoptosis of syncytiotrophoblasts, can hinder the secretion of trophoblast gonadotropin. These factors may be crucial mechanisms through which anti-annexin V antibodies influence embryo implantation upon binding and the results of pregnancy (41). Annexin A4 (*ANXA4*) is a member of the annexin family that belongs to the multigene family of calcium ion (Ca^2+^) and phospholipid-binding proteins. The gene encoding human *ANXA4* is located on chromosome 2q13.3. The C-terminal conserved domain contains four annexin repeats, each of which has a Ca^2+^-binding site and five *α*-helical structures, which can bind phospholipids in a calcium-dependent manner. The N-terminal domain is 12 amino acids long and determines the function of *ANXA4 in vivo*. *ANXA4* can regulate membrane permeability and membrane transport, participate in cell growth and apoptosis, increase tumor invasion and promote antitumor drug resistance (24). An increasing number of studies have shown that *ANXA4* is highly expressed in various clinical tumors and is an indicator of tumor development, invasion, chemoresistance, and poor outcomes in cancer patients (42–45). For example, *ANXA4* is upregulated and translocated to the nucleus in ovarian clear cell carcinoma and colorectal cancer (44, 45), and the knockdown of *ANXA4* weakens the migration and invasion of ovarian cancer and breast cancer cells (43, 46). In addition, several studies have shown that *ANXA4* expression is significantly increased in the oviductal fluid of pregnant mares and bovines, and in the uterine endometrium during early pregnancy in pigs, *ANXA4* has been identified as an embryo-interacting protein originating from oviductal fluid (47–49). Additionally, the *ANXA4* transcript in the human endometrium is significantly upregulated during the menstrual secretory phase compared with the proliferative phase (50). Previous studies reported that elevated *ANXA4* mRNA levels are correlated with an increasing level of progesterone (51), and might play a crucial role in the receptive process (52). Proportionately, proteomic *PLCD4* (p.L696P) analyses of human placental tissues have shown that *ANXA4* expression is downregulated in preeclampsia (PE) placentas and PE placenta-derived extravillous cytotrophoblasts compared with the expression in normal placentas (53). Additionally, *ANXA4* overexpression alleviated rat PE progression, accompanied by increases in the expression of PI3K, p-Akt, and p-eNOS in rat placentas, which indicated that *ANXA4* may promote trophoblast invasion through the PI3K/Akt/eNOS pathway (53). Furthermore, the *ANXA4* gene may play an important role in pregnancy, but there are currently no reports on whether it is involved in the occurrence of miscarriage.

RSA is a multifactorial disease, the exact causes of which are still unknown. Environmental, maternal, and genetic factors have been shown to contribute to this condition (54). WES is widely used to detect genetic variations associated with human diseases, including RSA. In recent years, using WES, studies in families with inherited recurrent pregnancy loss (RPL) confirmed the associations of a number of gene variations with RPL, including *IFT122* (p.V553G, p.S373F, p.W7C, p.G546R)*, ASIC5* (p.R227I)*, DYNC2H1* (p.Y2016C, p.D2184V)*, ALOX15* (p.Y139C, p.T560M)*, FKBP4* (p.A16E, p.N125S, p.Q381L, p.R399Q)*, PLCD4* (p.L696P) and *OSBPL5* (p.G385R) (55–59). The compromised expression of FKBP4, resulting from the increased expression of miR-29c, leads to impaired progesterone signaling and defects in decidualization (60). This may contribute to the development of endometriosis and infertility. In addition, studies in unrelated RSA patients and controls revealed potentially harmful gene variants, including *APP* (p.K510N), *FN1* (p.M1874T), *KDR* (p.D814N), *POLR2B* (p.G136C), *ITGB1* (p.Y219H), *PLK1* (p.A404S), *COL4A2* (p.H1603P), *LAMA4* (p.D1053G), *FOXA2* (p.Y420X), *FGA* (p.A762V), *F13A1* (p.Q401X, p.R612C), *KHDC3L* (p.146_156del), *ANXA5* (p.G317R), *DNMT1* (p.G876R), *THBS1* (p.N700S), and *MSH2* (p.L390F) (11, 61). Earlier research has offered significant understanding regarding the function of human KHDC3L in the modulation of homologous recombination (HR) repair, the activation of PARP1, and the maintenance of genome stability, while also recognizing human KHDC3L as an emerging risk gene associated with RPL (62). Previous studies have demonstrated that reduced expression of ANXA5, associated with the M2 haplotype, increases the risk of vascular thrombosis during pregnancy, thereby increasing the occurrence of RPL, primary maternal peripheral complications (PMPC), and recurrent implantation failure (RIF) (63). In the present study, a total of 325 patients with RSA and 941 local women without RSA as controls were included, and we identified specific gene variants that are pathogenic or possibly pathogenic through WES analysis. Among those variants, a novel *ANXA4* mutation (p.G8D) in 1 of the 325 patients with RSA (RSA-219) was detected. This mutation was not detected in either the 941 controls or public databases (dbSNP, 1,000 Genomes Project, ExAC and BGI in-house databases). The SIFT program prediction and structural modeling analysis show that this mutation is harmful. Furthermore, functional analyses revealed that this mutation could inhibit cell migration, invasion and adhesion. Moreover, despite appropriate fertility preservation treatment, the patient experienced another recurrent miscarriage in 2020 during the follow-up period. These results indicate that the *ANXA4* mutation p.G8D plays an important role in the pathogenesis of RSA. To the best of our knowledge, this is the first report revealing a novel *ANXA4* mutation that may be associated with RSA. Insights from these studies can help us better identify recurrent miscarriages. By discovering new mutations in the *ANXA4* gene that are associated with recurrent miscarriage, geneticists can work with clinicians to identify patients with deleterious variants in orthologous genes, thereby playing an active role in personalized medicine. However, the underlying mechanisms by which this mutation affects RSA need further investigation.

## 5 Conclusion

In conclusion, for the first time, we detected a novel *ANXA4* mutation (p.G8D) in 325 samples from women with RSA using WES analysis, this mutation was not detected in either 941 local control women or public databases. Furthermore, functional analyses revealed that this mutation could inhibit cell migration, invasion and adhesion. Taken together, our findings suggest that the *ANXA4* mutation p.G8D plays an important role in the pathogenesis of RSA and may contribute to its genetic diagnosis.

## Data Availability

The datasets presented in this study can be found in online repositories. The names of the repository/repositories and accession number(s) can be found in the article/supplementary material.

## Footnotes

^1^ http://www.ncbi.nlm.nih.gov/projects/snp/

^2^ www.1000genomes.org/

^3^ https://www.ncbi.nlm.nih.gov/gene/

^4^ http://www.expasy.org/

^5^ http://snpeff.sourceforge.net/SnpEff_manual.html

## References

[1] Practice Committee of the American Society for Reproductive Medicine. Electronic address aao. Definitions of infertility and recurrent pregnancy loss: a committee opinion. Fertil Steril. (2020) 113:533–5. doi: 10.1016/j.fertnstert.2019.11.025, PMID: 32115183

[2] Zhang LM Yang YN Zhang RX Luo L Tan JF Zhou L. Comparison of the etiological constitution of two and three or more recurrent miscarriage. Zhonghua Fu Chan Ke Za Zhi. (2018) 53:855–9. doi: 10.3760/cma.j.issn.0529-567x.2018.12.010 30585025

[3] Youssef A Vermeulen N Lashley E Goddijn M van der Hoorn MLP. Comparison and appraisal of (inter) national recurrent pregnancy loss guidelines. Reprod Biomed Online. (2019) 39:497–503. doi: 10.1016/j.rbmo.2019.04.008, PMID: 31182358

[4] Practice Committee of the American Society for Reproductive Medicine. Evaluation and treatment of recurrent pregnancy loss: a committee opinion. Fertil Steril. (2012) 98:1103–11. doi: 10.1016/j.fertnstert.2012.06.048 22835448

[5] Wu M Zhu Y Zhao J Ai H Gong Q Zhang J. Soluble costimulatory molecule sTim3 regulates the differentiation of Th1 and Th2 in patients with unexplained recurrent spontaneous abortion. Int J Clin Exp Med. (2015) 8:8812–9. PMID: 26309533 PMC4537953

[6] Popescu F Jaslow CR Kutteh WH. Recurrent pregnancy loss evaluation combined with 24-chromosome microarray of miscarriage tissue provides a probable or definite cause of pregnancy loss in over 90% of patients. Hum Reprod. (2018) 33:579–87. doi: 10.1093/humrep/dey021, PMID: 29538673

[7] Rodrigues VO Soligo A Pannain GD. Antiphospholipid antibody syndrome and infertility. Rev Bras Ginecol Obstet. (2019) 41:621–7. doi: 10.1055/s-0039-1697982 31658490

[8] Ticconi C Pietropolli A Di Simone N Piccione E Fazleabas A. Endometrial immune dysfunction in recurrent pregnancy loss. Int J Mol Sci. (2019) 20:5332. doi: 10.3390/ijms20215332, PMID: 31717776 PMC6862690

[9] di Simone N Castellani R Raschi E Borghi MO Meroni PL Caruso A. Anti-beta-2 glycoprotein I antibodies affect Bcl-2 and Bax trophoblast expression without evidence of apoptosis. Ann N Y Acad Sci. (2006) 1069:364–76. doi: 10.1196/annals.1351.034, PMID: 16855163

[10] D'Ippolito S Ticconi C Tersigni C Garofalo S Martino C Lanzone A. The pathogenic role of autoantibodies in recurrent pregnancy loss. Am J Reprod Immunol. (2020) 83:e13200. doi: 10.1111/aji.13200, PMID: 31633847

[11] Mou JT Huang SX Yu LL Xu J Deng QL Xie YS. Identification of genetic polymorphisms in unexplained recurrent spontaneous abortion based on whole exome sequencing. Ann Transl Med. (2022) 10:603. doi: 10.21037/atm-22-2179, PMID: 35722368 PMC9201170

[12] Claussnitzer M Cho JH Collins R Cox NJ Dermitzakis ET Hurles ME. A brief history of human disease genetics. Nature. (2020) 577:179–89. doi: 10.1038/s41586-019-1879-7, PMID: 31915397 PMC7405896

[13] Erdenee S Akhatayeva Z Pan C Cai Y Xu H Chen H. An insertion/deletion within the CREB1 gene identified using the RNA-sequencing is associated with sheep body morphometric traits. Gene. (2021) 775:145444. doi: 10.1016/j.gene.2021.145444 33484760

[14] Savoldi IR Ibelli AMG Cantao ME Peixoto JO Pires MP Mores MAZ. A joint analysis using exome and transcriptome data identifiescandidate polymorphisms and genes involved with umbilical hernia in pigs. BMC Genomics. (2021) 22:818. doi: 10.1186/s12864-021-08138-4, PMID: 34773987 PMC8590244

[15] Li C Ou R Hou Y Chen Y Wei Q Zhang L. Rare variant analysis of PTRHD1 in Parkinson's disease in the Chinese population. J Parkinsons Dis. (2022) 12:1917–20. doi: 10.3233/JPD-223337, PMID: 35848037

[16] Kolte AM Nielsen HS Moltke I Degn B Pedersen B Sunde L. A genome-wide scan in affected sibling pairs with idiopathic recurrent miscarriage suggests genetic linkage. Mol Hum Reprod. (2011) 17:379–85. doi: 10.1093/molehr/gar003, PMID: 21257601

[17] Karim S Jamal HS Rouzi A Ardawi MSM Schulten HJ Mirza Z. Genomic answers for recurrent spontaneous abortion in Saudi Arabia: an array comparative genomic hybridization approach. Reprod Biol. (2017) 17:133–43. doi: 10.1016/j.repbio.2017.03.003, PMID: 28431992

[18] Pereza N Ostojic S Kapovic M Peterlin B. Systematic review and meta-analysis of genetic association studies in idiopathic recurrent spontaneous abortion. Fertil Steril. (2017) 107:150–159.e2. doi: 10.1016/j.fertnstert.2016.10.007, PMID: 27842992

[19] Loizidou EM Kucherenko A Tatarskyy P Chernushyn S Livshyts G Gulkovskyi R. Risk of recurrent pregnancy loss in the Ukrainian population using a combined effect of genetic variants: a case-control study. Genes (Basel). (2021) 12:64. doi: 10.3390/genes12010064, PMID: 33466305 PMC7824779

[20] Povysil G Petrovski S Hostyk J Aggarwal V Allen AS Goldstein DB. Rare-variant collapsing analyses for complex traits: guidelines and applications. Nat Rev Genet. (2019) 20:747–59. doi: 10.1038/s41576-019-0177-4, PMID: 31605095

[21] Gourhant L Bocher O De Saint ML Ludwig TE Boland A Deleuze JF. Whole exome sequencing, a hypothesis-free approach to investigate recurrent early miscarriage. Reprod Biomed Online. (2021) 42:789–98. doi: 10.1016/j.rbmo.2021.01.008, PMID: 33658156

[22] Smail C Ferraro NM Hui Q Durrant MG Aguirre M Tanigawa Y. Integration of rare expression outlier-associated variants improves polygenic risk prediction. Am J Hum Genet. (2022) 109:1055–64. doi: 10.1016/j.ajhg.2022.04.015, PMID: 35588732 PMC9247823

[23] Arii Y Butsusihta K Fukuoka S. Role of calcium-binding sites in calcium-dependent membrane association of annexin A4. Biosci Biotechnol Biochem. (2015) 79:978–85. doi: 10.1080/09168451.2014.1003131, PMID: 25649809

[24] Yao H Sun C Hu Z Wang W. The role of annexin A4 in cancer. Front Biosci (Landmark Ed). (2016) 21:949–57. doi: 10.2741/4432, PMID: 27100483

[25] Ponnampalam AP Rogers PA. Cyclic changes and hormonal regulation of annexin IV mRNA and protein in human endometrium. Mol Hum Reprod. (2006) 12:661–9. doi: 10.1093/molehr/gal075, PMID: 16954445

[26] Li H Durbin R. Fast and accurate short read alignment with burrows-wheeler transform. Bioinformatics. (2009) 25:1754–60. doi: 10.1093/bioinformatics/btp324, PMID: 19451168 PMC2705234

[27] Van der Auwera GA Carneiro MO Hartl C Poplin R Del Angel G Levy-Moonshine A. From fast Q data to high confidence variant calls: the genome analysis toolkit best practices pipeline. Curr Protoc Bioinformatics. (2013) 43:11.10.1–11.10.33. doi: 10.1002/0471250953.bi1110s43 PMC4243306 25431634

[28] Yang H Wang K. Genomic variant annotation and prioritization with ANNOVAR and wANNOVAR. Nat Protoc. (2015) 10:1556–66. doi: 10.1038/nprot.2015.105, PMID: 26379229 PMC4718734

[29] Sherry ST Ward MH Kholodov M Baker J Phan L Smigielski EM. Db SNP: the NCBI database of genetic variation. Nucleic Acids Res. (2001) 29:308–11. doi: 10.1093/nar/29.1.308 11125122 PMC29783

[30] Lek M Karczewski KJ Minikel EV Samocha KE Banks E Fennell T. Analysis of protein-coding genetic variation in 60, 706 humans. Nature. (2016) 536:285–91. doi: 10.1038/nature19057, PMID: 27535533 PMC5018207

[31] Greening DW Nguyen HP Evans J Simpson RJ Salamonsen LA. Modulating the endometrial epithelial proteome and secretome in preparation for pregnancy: the role of ovarian steroid and pregnancy hormones. J Proteome. (2016) 144:99–112. doi: 10.1016/j.jprot.2016.05.026 27262222

[32] Zhang S Lin H Kong S Wang S Wang H Wang H. Physiological and molecular determinants of embryo implantation. Mol Asp Med. (2013) 34:939–80. doi: 10.1016/j.mam.2012.12.011, PMID: 23290997 PMC4278353

[33] Brosens JJ Pijnenborg R Brosens IA. The myometrial junctional zone spiral arteries in normal and abnormal pregnancies: a review of the literature. Am J Obstet Gynecol. (2002) 187:1416–23. doi: 10.1067/mob.2002.127305, PMID: 12439541

[34] Ticconi C Di Simone N Campagnolo L Fazleabas A. Clinical consequences of defective decidualization. Tissue Cell. (2021) 72:101586. doi: 10.1016/j.tice.2021.101586, PMID: 34217128

[35] Gellersen B Reimann K Samalecos A Aupers S Bamberger AM. Invasiveness of human endometrial stromal cells is promoted by decidualization and by trophoblast-derived signals. Hum Reprod. (2010) 25:862–73. doi: 10.1093/humrep/dep468 20118488

[36] Ren L Liu YQ Zhou WH Zhang YZ. Trophoblast-derived chemokine CXCL12 promotes CXCR4 expression and invasion of human first-trimester decidual stromal cells. Hum Reprod. (2012) 27:366–74. doi: 10.1093/humrep/der395 22114110

[37] Weimar CH Macklon NS Post Uiterweer ED Brosens JJ Gellersen B. The motile and invasive capacity of human endometrial stromal cells: implications for normal and impaired reproductive function. Hum Reprod Update. (2013) 19:542–57. doi: 10.1093/humupd/dmt025, PMID: 23827985

[38] Murata H Tanaka S Okada H. The regulators of human endometrial stromal cell Decidualization. Biomol Ther. (2022) 12:1275. doi: 10.3390/biom12091275, PMID: 36139114 PMC9496326

[39] Hu J Chen L Ruan J Chen X. The role of the annexin a protein family at the maternal-fetal interface. Front Endocrinol (Lausanne). (2024) 15:1314214. doi: 10.3389/fendo.2024.1314214, PMID: 38495790 PMC10940358

[40] Garrido-Gomez T Quiñonero A Dominguez F Rubert L Perales A Hajjar KA. Preeclampsia: a defect in decidualization is associated with deficiency of Annexin A2. Am J Obstet Gynecol. (2020) 222:376.e1–e17. doi: 10.1016/j.ajog.2019.11.1250, PMID: 31738896

[41] Di Simone N Castellani R Caliandro D Caruso A. Monoclonal anti-annexin V antibody inhibits trophoblast gonadotropin secretion and induces syncytiotrophoblast apoptosis. Biol Reprod. (2001) 65:1766–70. doi: 10.1095/biolreprod65.6.1766, PMID: 11717139

[42] Konopka-Postupolska D Clark G Hofmann A. Structure, function and membrane interactions of plant annexins: an update. Plant Sci. (2011) 181:230–41. doi: 10.1016/j.plantsci.2011.05.013, PMID: 21763533

[43] Mogami T Yokota N Asai-Sato M Yamada R Koizume S Sakuma Y. Annexin A4 is involved in proliferation, chemo-resistance and migration and invasion in ovarian clear cell adenocarcinoma cells. PLoS One. (2013) 8:e80359. doi: 10.1371/journal.pone.0080359, PMID: 24244679 PMC3823662

[44] Liu J Wang H Zheng M Deng L Zhang X Lin B. P 53 and ANXA4/NF-kappaB p50 complexes regulate cell proliferation, apoptosis and tumor progression in ovarian clear cell carcinoma. Int J Mol Med. (2020) 46:2102–14. doi: 10.3892/ijmm.2020.4757, PMID: 33125094 PMC7595666

[45] Peng Y Zhang Z Zhang A Liu C Sun Y Peng Z. Membrane-cytoplasm translocation of annexin A4 is involved in the metastasis of colorectal carcinoma. Aging (Albany NY). (2021) 13:10312–25. doi: 10.18632/aging.202793, PMID: 33761465 PMC8064178

[46] Li L Zhang R Liu Y Zhang G. ANXA4 activates JAK-STAT3 signaling by interacting with ANXA1 in basal-like breast Cancer. DNA Cell Biol. (2020) 39:1649–56. doi: 10.1089/dna.2020.5570, PMID: 32552056

[47] Smits K Willems S Van Steendam K Van De Velde M De Lange V Ververs C. Proteins involved in embryo-maternal interaction around the signalling of maternal recognition of pregnancy in the horse. Sci Rep. (2018) 8:5249. doi: 10.1038/s41598-018-23537-6, PMID: 29588480 PMC5869742

[48] Banliat C Tsikis G Labas V Teixeira-Gomes AP Com E Lavigne R. Identification of 56 proteins involved in embryo-maternal interactions in the bovine oviduct. Int J Mol Sci. (2020) 21:466. doi: 10.3390/ijms21020466, PMID: 31940782 PMC7013689

[49] Pierzchala D Liput K Korwin-Kossakowska A Ogluszka M Polawska E Nawrocka A. Molecular characterisation of uterine endometrial proteins during early stages of pregnancy in pigs by MALDI TOF/TOF. Int J Mol Sci. (2021) 22:6720. doi: 10.3390/ijms22136720, PMID: 34201586 PMC8267828

[50] Ponnampalam AP Weston GC Trajstman AC Susil B Rogers PA. Molecular classification of human endometrial cycle stages by transcriptional profiling. Mol Hum Reprod. (2004) 10:879–93. doi: 10.1093/molehr/gah121, PMID: 15501903

[51] Talbi S Hamilton AE Vo KC Tulac S Overgaard MT Dosiou C. Molecular phenotyping of human endometrium distinguishes menstrual cycle phases and underlying biological processes in normo-ovulatory women. Endocrinology. (2006) 147:1097–121. doi: 10.1210/en.2005-1076, PMID: 16306079

[52] Mirkin S Arslan M Churikov D Corica A Diaz JI Williams S. In search of candidate genes critically expressed in the human endometrium during the window of implantation. Hum Reprod. (2005) 20:2104–17. doi: 10.1093/humrep/dei051, PMID: 15878921

[53] Xu Y Sui L Qiu B Yin X Liu J Zhang X. ANXA4 promotes trophoblast invasion via the PI3K/Akt/eNOS pathway in preeclampsia. Am J Physiol Cell Physiol. (2019) 316:C481–91. doi: 10.1152/ajpcell.00404.2018, PMID: 30673304

[54] Guo W Zhu X Yan L Qiao J. The present and future of whole-exome sequencing in studying and treating human reproductive disorders. J Genet Genomics. (2018) 45:517–25. doi: 10.1016/j.jgg.2018.08.004, PMID: 30391409

[55] Tsurusaki Y Yonezawa R Furuya M Nishimura G Pooh RK Nakashima M. Whole exome sequencing revealed biallelic IFT122 mutations in a family with CED1 and recurrent pregnancy loss. Clin Genet. (2014) 85:592–4. doi: 10.1111/cge.12215 23826986

[56] Filges I Manokhina I Penaherrera MS McFadden DE Louie K Nosova E. Recurrent triploidy due to a failure to complete maternal meiosis II: whole-exome sequencing reveals candidate variants. Mol Hum Reprod. (2015) 21:339–46. doi: 10.1093/molehr/gau112, PMID: 25504873 PMC4381034

[57] Qiao Y Wen J Tang F Martell S Shomer N Leung PC. Whole exome sequencing in recurrent early pregnancy loss. Mol Hum Reprod. (2016) 22:364–72. doi: 10.1093/molehr/gaw008, PMID: 26826164 PMC4847612

[58] Demetriou C Chanudet E GOSgene Joseph A Topf M Thomas AC. Exome sequencing identifies variants in FKBP4 that are associated with recurrent fetal loss in humans. Hum Mol Genet. (2019) 28:3466–74. doi: 10.1093/hmg/ddz203, PMID: 31504499

[59] Al Qahtani NH AbdulAzeez S Almandil NB Fahad Alhur N Alsuwat HS Al Taifi HA. Whole-genome sequencing reveals Exonic variation of ASIC5 gene results in recurrent pregnancy loss. Front Med (Lausanne). (2021) 8:699672. doi: 10.3389/fmed.2021.699672, PMID: 34395479 PMC8363113

[60] Joshi NR Miyadahira EH Afshar Y Jeong JW Young SL Lessey BA. Progesterone resistance in endometriosis is modulated by the altered expression of Micro RNA-29c and FKBP4. J Clin Endocrinol Metab. (2017) 102:141–9. doi: 10.1210/jc.2016-2076, PMID: 27778641 PMC5413101

[61] Xiang H Wang C Pan H Hu Q Wang R Xu Z. Exome-sequencing identifies novel genes associated with recurrent pregnancy loss in a Chinese cohort. Front Genet. (2021) 12:746082. doi: 10.3389/fgene.2021.746082, PMID: 34925444 PMC8674582

[62] Zhang W Chen Z Zhang D Zhao B Liu L Xie Z. KHDC3L mutation causes recurrent pregnancy loss by inducing genomic instability of human early embryonic cells. PLoS Biol. (2019) 17:e3000468. doi: 10.1371/journal.pbio.3000468, PMID: 31609975 PMC6812846

[63] Peng L Yang W Deng X Bao S. Research progress on ANXA5 in recurrent pregnancy loss. J Reprod Immunol. (2022) 153:103679. doi: 10.1016/j.jri.2022.103679, PMID: 35964539

